# Editorial: Bioactive materials for disease diagnosis and therapy

**DOI:** 10.3389/fbioe.2024.1403980

**Published:** 2024-04-05

**Authors:** Yuce Li, Jing He, Jixiu Liu, Mingqiang Li, Jie Tang, Dan Shao

**Affiliations:** ^1^ College of Life Science and Health, Wuhan University of Science and Technology (WUST), Wuhan, China; ^2^ Laboratory of Biomaterials and Translational Medicine, Center for Nanomedicine, The Third Affiliated Hospital, Sun Yat-sen University, Guangzhou, China; ^3^ Monash Institute of Pharmaceutical Sciences, Monash University, Parkville, VIC, Australia; ^4^ School of Biomedical Sciences and Engineering, Guangzhou International Campus, South China University of Technology, Guangzhou, China

**Keywords:** bioactive materials, drug delivery, tissue engineering, regenerative medicine, disease diagnostics

Traditional biomaterials such as drug carriers and tissue engineering scaffolds are often biologically inert to avoid unnecessary concerns regarding biocompatibility. Bioactive materials are a class of materials that possess intrinsic biological activity, allowing them to interact with organs, tissues, or cells in the microenvironment of diseases, thereby regulating biological functions or enhancing the therapeutic effect of intervention. The emergence of bioactive materials has provided a new paradigm for the treatment and diagnosis of diseases. This Research Topic focuses on bioactive materials for disease diagnosis and treatment, with a total of 16 papers published, including seven review papers and nine original research papers. These papers were authored by 118 researchers from universities, hospitals, research institutes, and companies around the world, and have attracted 25.1 thousand views, 5.5 thousand downloads, and 15 citations from researchers worldwide as of 20 March 2024. Certainly, this Research Topic has undoubtedly served as a valuable platform for researchers to exchange advancements in the field of biologically active materials.

The seven review papers provided by this Research Topic summarize the research progress of bioactive materials such as nanomaterials, nanobiosensors, and dental implants, outlining their applications in the diagnosis and treatment of diseases such as ischemic stroke, bone and cartilage repair, bacterial infections, tuberculosis, and urinary tissue engineering. Zhan et al. introduced the advancements in stimulus-responsive nanomedicines for treating ischemic stroke. Duan et al. outlined the utilization of bioactive materials and innovative technologies for urinary regeneration and tissue engineering. In the field of dental implants, Wang et al. comprehensively reviewed the progress in biomimetic structures and associated signal pathways regarding the surface modification methods and emerging biomaterials to accelerate osseointegration. Yang et al. systematically introduced the current research status of functional nanobiosensors for diagnosis of tuberculosis. In the field of combating bacterial infections, Chen et al. specifically introduced bioactive materials for targeted delivery of antibiotics from the aspects of drug delivery and endogenous biological functions. Yu et al. introduced the research progress of graphene and its derivatives in the fields of bone tissue engineering and cartilage tissue engineering, and prospected the possible direction of graphene-based materials in orthopedics. Particularly, Fernández-Gómez et al. focused on the latest nanotechnology developed by Spanish authors for disease treatments during the period from 2017 to 2022, and outlined the future trends and directions of nanomedical research.

Since the dawn of human civilization, we have grappled with the challenge of malignant tumors, while they persisted as a significant threat to human health and life. Treatments that leverage the generation of reactive oxygen species (ROS) within tumor cells to induce tumor cell death, including photodynamic therapy, chemotherapy, and so on, represent a class of recently developed therapies with good therapeutic effects and fewer side effects. Luo et al. encapsulated the photosensitizer chlorin e6 (Ce6) within albumin nanocages to fabricate Ce6-albumin nanoparticles (CA-NPs) using a well-defined nanoprecipitation method. These nanoparticles exhibit enhanced photostability, photoreactivity, and remarkable resistance towards photobleaching compared to free Ce6. Animal experiments have demonstrated the efficient accumulation of these nanoparticles in bladder cancer, leading to significant tumor treatment efficacy upon light exposure. Moreover, these nanoparticles effectively induced apoptosis in fresh human bladder tumor tissue samples. In another study, Zhang et al. introduced a novel nanocatalyst based on non-oxidized MXene-Ti_3_C_2_Tx quantum dots. Within cancer cells, this nanocatalyst facilitates the Fenton reaction, converting hydrogen peroxide into highly reactive hydroxyl radicals (•OH). Consequently, it induces ferroptosis in tumor cells through lipid peroxidation and mitochondrial dysfunction. Moreover, Zhang et al. utilized cancer cell membrane (CM)-coated mesoporous silica nanoparticles (MSNs) to load a two-photon fluorescence probe (TPFP), constructing a tumor-targeted bioactive nanosensor. This sensor enables the monitoring of hydrogen peroxide levels generated within tumors using two-photon microscopy and predicts the prognosis of chemotherapy based on hydrogen peroxide content. Photothermal therapy and gas therapy are two types of non-invasive treatments that kill tumor cells by generating heat or releasing gases such as carbon monoxide/nitric oxide locally in the tumor. Zuo et al. loaded nitric oxide donors into gold nanoparticles and investigated their applications in radiotherapy sensitization, photothermal therapy and nitric oxide gas therapy. Through the combination of near-infrared light and X-ray irradiation, these nanoparticles released a large amount of heat, ROS, and nitric oxide locally in the tumor. It is noteworthy that the reaction between ROS and nitric oxide produces more toxic reactive nitrogen species (RNS). This synergistic effect leads to excellent anti-tumor therapeutic effects.

Bacterial infections pose a significant obstacle during the process of skin tissue regeneration and represent a serious threat to human health. Despite the development of numerous antibiotics to combat bacterial infections, the emergence of superbugs has rendered these antibiotics ineffective. Bioactive materials for antimicrobial purposes have been widely utilized in various aspects of daily life. Liu et al. prepared a complex of Cu^2+^ and MXene (Cu(II)@MXene) and incorporated it into hyaluronic acid hydrogel to fabricate antibacterial dressings. These dressings facilitate easy application to wounds of various shapes owing to their rapid adhesion, self-healing, and injectability properties. The Cu(II)@MXene significantly accelerated the healing of infected wounds by serving as a photothermal antibacterial barrier, eliminating ROS generated during wound healing and promoting vascular regeneration. Bao et al. synthesized a bioactive antibacterial material, Ag-TiO_2_/ZIF-8, using a solvothermal method. They achieved excellent antibacterial effects against *E. coli* and *B. subtilis* by controlling the ratio of Ag-TiO_2_. Moreover, the prepared material demonstrated good stability and durability, and the antibacterial activity remained effective for over 5 months.

Tissue regeneration and repair provide crucial opportunities for the treatment of various diseases and injuries. Intracranial stents serve to support blood vessels, restore cerebral blood flow, and prevent ischemia and damage to brain tissue, playing a significant role in the treatment and postoperative repair of cerebrovascular diseases. Bi et al. incorporated a bioactive compound derived from *Salvia miltiorrhiza*, salvianolic acid B (SALB), into chitosan and immobilized it onto a nickel-titanium alloy plate using dopamine, thereby creating a bioactive coating. Continuous release of SALB was observed within 28 days after application. This bioactive layer effectively inhibited the proliferation, adhesion, and migration of smooth muscle cells, thereby preventing the occurrence of neointimal hyperplasia and restenosis. Osteogenesis and the restoration of bone function are closely associated with the generation of nerves and blood vessels. Han et al. investigated the characteristics of sensory nerves and neovascularization during *in situ* osteogenesis, examining their relationship with neurovascular networks, mineralization, and their biological regulators. The findings revealed that during osteogenesis, the expression of semaphorin 3A (Sema3A) increased, initiating the appearance of sensory fibers followed by vascular and bone formation. Furthermore, the occurrence of innervation and vascularization showed temporal and spatial correlation. Thus, Sema3A emerges as a potential target for regulating bone formation.

The growing emphasis on healthy living has spurred rapid developments in beauty and anti-aging treatments over recent years. While stem cells possess excellent therapeutic potential for skin rejuvenation, their safety concerns constrain their widespread application. Extracellular vesicles derived from stem cells contain biological contents similar to those of stem cells and exhibit therapeutic efficacy comparable to stem cells in anti-aging and skin repair. Ye et al. isolated exosomes from mesenchymal stem cells (hMSC-Exo) and investigated their potential in treating sensitive skin. *In vitro* experiments revealed that hMSC-Exo significantly boosted the proliferation and migration of human fibroblasts. Importantly, the authors conducted a clinical trial, demonstrating that twice-daily application of hMSC-Exo for 28 days markedly reduced skin sensitivity to lactic acid-induced sting and decreased skin sebum production. These findings suggest that hMSC-Exo exerts a substantial alleviating effect on symptoms in individuals with sensitive skin.

In summary, this Research Topic included a series of excellent research papers regarding bioactive materials and their applications in disease diagnosis and therapy. It may help the researchers around the world to share their opinions in this field. It is foreseeable that this field will become an important branch in biomaterials and play more important roles in disease treatment and diagnoses.

